# Novel Haloalkaliphilic Nitrile-Degrading Bacteria From Soda Lake Soil of Rift Valley Kenya

**DOI:** 10.1155/tswj/3126129

**Published:** 2025-11-19

**Authors:** Meir Dayan Akinyi, Romano Mwirichia, Julius Mugweru, Njogu M. Kimani

**Affiliations:** ^1^Department of Biological Sciences, University of Embu, Embu, Kenya; ^2^Department of Physical Sciences, University of Embu, Embu, Kenya

**Keywords:** halophile bacteria, nitrilase, nitrile, nitrile hydratase, soda lakes

## Abstract

**Introduction:**

Nitrile biotransformation has garnered significant attention in recent years due to its widespread applications in various industries. This study is aimed at investigating the ability of haloalkaliphilic bacteria from extremely haloalkaline lakes in Kenya to produce unique nitrilases with nitrile-degrading potential.

**Methodology:**

A combination of enrichment and isolation was done on a mineral supplemented with either butyronitrile or isobutyronitrile, colorimetric assays, nesslerization method, Bertholet reaction, and molecular characterization.

**Results:**

Sixty bacterial isolates were recovered, of which 14 exhibited 6 nitrilase and 8 nitrile hydratase enzyme activities. The isolates, affiliated with *Bacillus agaradhaerens*, *Bacillus halodurans*, *Bacillus xiamenensis*, *Bacillus cellulosilyticus*, *Nesterenkonia alba*, *Nesterenkonia aethopica*, *Nesterenkonia* sp. YIM, *Alkalilimnicola* sp. AKP2, and *Alkalilimnicola haloduran* species, demonstrated optimal growth at pH 8.0–9.0, 5% salt concentration, and 28°C–40°C temperature. Notably, the nitrilase and nitrile hydratase enzymes exhibited optimal pH activity at 7.0–7.5.

**Conclusion:**

This study identifies novel bacterial isolates from Kenyan soda lakes with the ability to produce nitrilase and nitrile hydratase enzymes, which can be utilized for the hydrolysis of nitrile to carboxylic acid, ammonia, and amide.

**Significance and Impact of Study:**

The identification of bacterial strains capable of degrading nitriles into acids and amide compounds that are environmentally safe and beneficial to the green industry highlights a promising approach for mitigating the harmful effects of toxic nitriles in the environment. Biodegradation of nitriles by bacteria from soda lakes offers a sustainable solution to reduce ecological damage. Techniques such as enzymatic assays, colorimetric methods, the Berthelot reaction, and the two-step nesslerization method are essential for isolating and characterizing nitrile-degrading bacteria. The discovery of these biocatalysts not only advances green catalyst research but also holds significant potential for applications in organic synthesis, biotechnology, and environmental remediation.

## 1. Introduction

Hypersaline soda lakes are characterized by extreme pH, salt, and temperature conditions [1]. Despite these elevated conditions, diverse species of flora and fauna flourish [2]. A diverse group of organisms has been reported from these ecosystems [3]. Organisms adapted to extreme ecosystems usually produce novel biocatalysts not found in bacteria from a normal environment [4, 5]. Other organisms produce acid and antibiotics used to inhibit the growth of competitors [2, 6]. Therefore, there has been increased interest in the discovery of new biocatalysts from extremophilic microorganisms due to their unique properties and potential application in industrial biotechnology and research [7–10]. Various studies have shown that halophilic bacteria produce new natural compounds and enzymes that have not yet been isolated from the cultured halophilic bacterial population [11].

Nitrile is a major component of herbicide solvents commonly used in agricultural pest control in Kenya [12]. The use of nitriles as bulk herbicide solvents has increased their distribution in the environment and the need for their remediation. Horticultural commercial farms and industries near the soda lakes in Kenya use synthetic herbicides, which end up accumulating in the water [5]. These herbicides, which contain nitrile compounds, have significant adverse effects on aquatic life and humans [13]. Chemical hydrolysis of nitrile compounds is time and money consuming, and it results in large amounts of by-products like acids and amides [14]. Nitriles can also be hydrolyzed by nitrilase and nitrile hydratase enzymes to produce an acid and ammonia, which do not harm the environment [15]. Therefore, enzymatic hydrolysis of nitrile-containing compounds is an environmentally safe method of converting the nitrile to a useful carboxylic acid and an amide.

Nitrilase has been used in industry to produce glycolic acid, nicotinic acid, cyano hydroxybutyric acid, and mandelic acid [16]. The ability of nitrile enzymes to function at elevated pH, salt, and temperature makes them attractive for application in industrial processes. We therefore hypothesized that haloalkaliphilic bacteria from an extremely haloalkaline environment in Kenya could produce unique nitrilases. We report on the ability of bacteria recovered from Lake Sonachi, Elementaita, Magadi, and Bogoria in Kenya to produce the nitrilase and nitrile hydratase enzymes, which hydrolyze nitrile into an acid and an amide.

Although nitrile-degrading microorganisms have been reported in contaminated soils and industrial effluents, there is limited knowledge on haloalkaliphilic bacteria from soda lakes in Kenya with the capacity to produce nitrilase and nitrile hydratase enzymes. Previous studies from Kenyan soda lakes have focused on lipases, esterases, and other hydrolases, but the potential for nitrile degradation in these extreme environments remains largely unexplored. This gap is significant because enzymes from haloalkaliphiles are often more robust and better suited for industrial biocatalysis under harsh conditions. Therefore, this study is aimed at isolating and characterizing novel nitrile-degrading bacteria from Kenyan soda lakes, evaluating their enzymatic activities, and assessing their potential applications in green chemistry and industrial biotechnology.

## 2. Methodology and Materials

### 2.1. Sample Collection and Nitrile-Degrading Bacterial Isolation

Lake Magadi is a hypersaline water body located at 2°S, 36°E at 600 m above sea level, 9.0–12.5 pH, 35% salt concentrations, and 22°C–37°C temperatures. Lake Sonachi is a volcanically formed alkaline crater lake in the intertropical zone, located 0°47⁣′ S 36°16⁣′ E, approximately 1670 m above sea level. Lake Elementaita measures 18 km^2^ and is a breeding and feeding ground for many threatened bird species. Lake Bogoria lies 60 km, 0°36 N and 36°04 E, north of the equator. Bogoria has 130 km^2^ surface area and is famous for its geysers; it has no surface outlet, so the water evaporates, becoming saline.

Water, grassland soil, and sediments were collected and transported in cool boxes to the laboratory. Isolation broth media used sterile lake water supplemented with butyronitrile (BN) or isobutyronitrile (IBN) as well as vitamin B_12_. In half a liter of sterile lake water, 2.5 mL of BN and/or IBN and 2 mL of vitamin B_12_ were added as shown in Table S1. The 5-mL aliquots of the enrichment broth were dispensed into 10-mL Hungate anaerobic tubes, inoculated with 0.1 g of soil or sediments from the four soda lakes, and incubated at 40°C for 21 days. The enriched cultures were fivefold serial diluted, and 100 *μ*L from 10^−5^, 10^−4^, and 10^−3^ was spread plated onto the enriched agar plates prepared as in Table S2 and incubated at 40°C for 30 days. Single colonies were transferred onto the same media in new plates by restreaking several times. Colonies were picked and purified, and the pure bacterial isolates were maintained in trypticase soy broth (TSB) for further characterization and analysis.

### 2.2. Screening for Production of Nitrilase and Nitrile Hydratase

Nitrilase and nitrile hydratase enzymes were screened by subculturing the isolates on freshly prepared TSB medium supplemented with either BN or IBN and incubating the tubes at 37°C and 115 rpm. According to Sahu et al. [17], colorimetric assays were used to screen for nitrile enzymes, and detection was done using bromothymol blue pH indicator dye. The screening was based on the color change of the indicator during the hydrolysis of BN and IBN. The reaction mixture, in triplicate, consisted of 200 *μ*L TSB medium, 10 *μ*L of 1% indicator, 10 *μ*L substrate, and 20 *μ*L cell suspension, and the microplates were incubated at 30°C for 18 h at 115 rpm. The controls contained *Escherichia coli* (negative) and a blank (control) containing TSB medium.

In order to distinguish nitrile hydratase from nitrilase-producing bacteria, ammonia was measured by nesslerization, hydrochloric acid, or copper sulfate method. We used 96-well plates with a mixture of 400 *μ*L TSB medium, 20 *μ*L of 0.01% substrates, and 40 *μ*L of cell suspension in duplicate and incubated at 30°C and 115 rpm for 18 h. Three different control tubes contained *E. coli* (negative control), BN, and IBN substrates (positive control). To detect ammonia production, 100 *μ*L of Nessler's reagent was added, and the samples were observed for any color change [17–19], and absorbance was measured using a spectrophotometer at 540 nm with ammonium as a calibrator.

### 2.3. Screening for Nitrile Hydratase and Nitrilase Activity

We quantified nitrilase and nitrile hydratase enzyme production as demonstrated by Sahu et al. [17] and Santoshkumar et al. [20]. Minimal broth containing glycerol, peptone, malt extract, and yeast extract was used to cultivate the isolates and incubated for 36 h at 30°C and 115 rpm to make the preculture. Production minimal medium supplemented with 1% BN or IBN and preculture was incubated in 15 mL for 48 h at 30°C and 115 rpm. Using centrifugation at 32,000 rpm, cells were harvested for 10 min at 4°C. Then, 10 Mm potassium phosphate buffer was used for washing, and the cells were resuspended in the same buffer. The activity of nitrile hydratase was determined by spectrophotometry using a 5.3 mL reaction mixture and incubated for an hour at 10°C and 115 rpm. Uninoculated tubes were included as controls. Chilled 0.1 N HCL at 5.3 mL was used to halt the reaction. Formation of the amide was analyzed by measuring absorbance at OD_540_ wavelength [17, 20]. Nitrilase activity was analyzed in a 2 mL reaction mixture (1.7 mL 10 Mm potassium phosphate buffer pH 7.4, bacterial cells 200 *μ*L, 1% BN or IBN, and 100 *μ*L) while the control tube contained heat-denatured cells. The reaction was incubated for 45 min at 30°C and 115 rpm. Bertholet reaction (phenol–acetone, sodium nitroprusside, and sodium hypochlorite) 0.2 mL of each was added and incubated for 15 min at 27°C to stop the reaction, and 0.6 mL of distilled water was used to dilute the sample. Ammonia released was analyzed by measuring absorbance at 540 nm with ammonia as a calibrator ([17]; Santoshkumar et al. [20]).

### 2.4. Physiological and Morphological Characterization of Bacterial Isolates

The bacterial isolates were analyzed for their ability to tolerate various pH levels (5.0, 6.0, 7.0, 8.0, 9.0, 10.0, 11.0, 12.0, and 13.0). Similarly, the isolates were analyzed for their ability to tolerate various salt concentrations (0%, 5%, 10%, 15%, 20%, 25%, 30%, and 35%) and temperature (28°C, 35°C, 45°C, 55°C, and 65°C). The capability to tolerate various physiological conditions was assessed by measuring OD_600_ after 24 h, and the results were represented in a graphical form. Morphological characterization of the isolates was performed by checking the color, elevation, shape, and form. The characterization was performed according to standard microbiological techniques as described by Cappuccino et al. [21].

### 2.5. Molecular Characterization of Bacteria Using 16S rRNA

DNA was extracted from bacterial isolates cultivated in 5 mL TSB at 37°C for 24 h. The extraction process followed the phenol–chloroform protocol outlined by Kiplimo et al. [9]. Subsequently, the 16S rRNA gene was amplified using the universal bacterial primer pair 8F and 1492R as detailed by Kiplimo et al. [9]. Amplification was carried out on a Sure Cycle 8800 (Agilent Technologies) in a 50 *μ*L reaction volume containing PCR water, polymerase buffer, primers, Taq polymerase, and genomic DNA. The PCR conditions included an initial denaturation step at 95°C for 5 min, followed by 36 cycles of denaturation (96°C for 1 min), annealing (53°C for 1 min), and extension (72°C for 1 min), with a final extension step at 72°C for 5 min. The resulting amplicons were separated on a 1% agarose gel in TAE buffer and visualized under UV light after staining with a fluorescent dye [22]. Purification of the amplified fragments was carried out using the QIAquick PCR purification Kit (Qiagen, Germany) according to the manufacturer's protocol. Subsequent sequencing of the PCR products was performed at Inqaba Biotech, South Africa, using the same universal primers. Gene sequences obtained from the isolates were edited using Chromas Lite and compared to public databases through the Basic Local Alignment Search Tool (BLAST) on the National Center for Biotechnology Information (NCBI) website (http://www.ncbi.nih.gov). The alignment was conducted using ClustalW software, and phylogenetic analyses were performed using the molecular evolutionary genetics analysis MEGA 11 [23] with the neighbor-joining method [24].

## 3. Results

As shown in Table S3 (morphological characterization of the bacterial isolates based on BN and IBN substrates), 60 bacterial isolates were recovered from the soil samples collected from the four Kenyan soda lakes on the enrichment media supplemented with BN and IBN, as summarized in Tables S1 (enrichment broth preparation per liter of sterile lake water) and Table S2 (bacteriological agar plate preparation). The colors of the isolates were yellow, white, and cream white. The isolate elevation was raised, concave, and flat growing on the media, and some were growing away from the streaking line. The colony shapes were rod and single, paired, chained, and clustered in arrangement (see Table S4 [the Gram staining results of the isolates that showed nitrilase and nitrile hydratase activity on indicator screening. All isolates BN01-IBN14 are rod shaped with IBN14, BN07, and BN05 are Gram negative while BN01, BN02, BN03, BN04, BN06, BN08, BN09, BN10, IBN11, IBN12, and IBN13 are Gram positive]). Production of nitrilase and nitrile hydratase enzymes was detected by color change in the bromothymol blue indicator at pH 7.4 (Table S5 [the colorimetric results of isolate BN01-IBN14. Nitrilase positive BN01, BN02, BN05, BN09, IBN11, and IBN12; nitrile hydratase positive BN03, BN04, BN06, BN07, BN08, BN10, IBN13, and IBN14; and controls; *E. coli* negative control; and IBN and BN positive controls]). Quantification of these enzymes was done by nesslerization, hydrochloric acid, copper sulfate, and Bertholet reaction method. Absorbance was measured spectrophotometrically at 540 nm for nitrilase and nitrile hydratase enzymes. In the presence of a carboxylic acid, the indicator for nitrile hydratase enzyme changed from blue to yellow, while blue to green in the presence of nitrilase (Table S6A [the confirmatory test for nitrilase and nitrile hydratase enzyme using Nessler's reagent. From BN1 to IBN14. Nitrilase positive BN01, BN02, BN05, BN09, IBN11, and IBN12; nitrile hydratase positive BN03, BN04, BN06, BN07, BN08, BN10, IBN13, and IBN14; and controls; *E. coli* negative control; and blanks of BN and IBN positive controls]).

### 3.1. Quantitative Screening for Nitrile-Degrading Enzymes

Eight bacterial isolates (BN03, BN04, BN06, BN07, BN08, BN10, IBN13, and IBN14) were positive for nitrile hydratase activity (Figure S1, quantitative analysis of nitrile hydratase producing bacteria [BN3, BN4, BN6, BN7, BN8, BN10, IBN13, and IBN14] with turbid color due to production of amide), whereas six (BN01, BN02, BN05, BN09, IBN11, and IBN12) were positive for nitrilase (Figure S2, quantitative analysis of nitrilase. Nitrilase-producing bacteria [BN1, BN2, BN5, BN9, IBN11, and IBN12] with blue color change due to production of carboxylic acid indicating presences of nitrilase enzyme), as shown in Table S5 (the colorimetric results of isolate BN01-IBN14. Nitrilase positive BN01, BN02, BN05, BN09, IBN11, and IBN12; nitrile hydratase positive BN03, BN04, BN06, BN07, BN08, BN10, IBN13, and IBN14, and controls; *E. coli* negative control; and IBN and BN positive control). In the nesslerization method used to quantify nitrile-degrading enzymes, BN02 and BN05 showed high nitrilase enzyme activity, while BN09 and BN01 showed lower nitrilase enzyme activity. IBN13 and BN10 isolates showed high nitrile hydratase activity, while BN06, BN03, BN07, BN08, and IBN14 showed lower nitrile hydratase activity (Figure S3). The pipeline was the same for other screening (Figure S3A [the confirmatory test for nitrilase and nitrile hydratase enzyme using Nessler's reagent. From BN01 to IBN14. Nitrilase positive BN01, BN02, BN05, BN09, IBN11, and IBN12; nitrile hydratase positive BN03, BN04, BN06, BN07, BN08, BN10, IBN13, and IBN14; and controls; *E. coli* negative control; and blanks of BN and IBN positive controls]; Table S6A [the confirmatory test for nitrilase and nitrile hydratase enzyme using Nessler's reagent. From BN1 to IBN14. Nitrilase positive BN01, BN02, BN05, BN09, IBN11, and IBN12; nitrile hydratase positive BN03, BN04, BN06, BN07, BN08, BN10, IBN13, and IBN14; and controls; *E. coli* negative control; and blanks of BN and IBN positive controls]). Isolates BN05 and BN02 showed high nitrilase enzyme activity, while BN02, BN09, IBN12, and BN11 showed low nitrilase enzyme activity with the Bertholet reaction method. BN03 and BN06 showed high nitrile hydratase activity, while IBN13, IBN14, BN10, and BN07 showed lower nitrile hydratase activity with the Bertholet reaction method (Table S6 [the summary on the quantification and qualification of nitrilase and nitrile hydratase enzymes by use of Nessler's reagent and nesslerization methods. Nitrilase positive BN01, BN02, BN05, BN09, IBN11, IBN12, nitrile hydratase positive BN03, BN04, BN06, BN07, BN08, BN10, IBN13, IBN14, and controls; *E. coli* negative control; and IBN and BN positive controls, ammonium chloride for calibration]). The bacterial isolates with nitrilase color change from colorless to blue, while the nitrile hydratase bacteria showed no color change.

### 3.2. Physiological Characterization of Bacterial Isolates

Despite originating from hypersaline soil, all the isolates exhibit growth across a broad pH spectrum. The optimum growth was observed at pH 8 and pH 9 and the least growth at pH 5 and pH 13. Isolates BN04 and BN07 showed optimum growth at pH 9 (Figure S4). All bacterial isolates tolerated different NaCl concentration levels, growth increased with increase in NaCl concentration from 0% to 10%, slightly decreasing towards 15% and 35% (Figure S5). The favorable growth was recorded at a 5% NaCl concentration. Isolates BN04 and BN08 had optimum growth at 5% NaCl concentration (Figure S5). These isolates also exhibited growth at a broad range of temperature, 28°C–65°C, and the favorable growth was recorded at 28°C–40°C (Figure S6). Growth was observed to be decreasing from 45°C to 65°C. Isolates BN01, BN02, BN07, BN08, BN10, IBN11, IBN12, IBN13, and IBN14 had the optimum growth at 40°C.

### 3.3. Molecular Characterization of Bacterial Isolates

The 16S rRNA gene sequence analysis showed that isolates BN02, BN06, BN08, BN09, BN10, IBN12, IBN13, IBN11, and IBN14 were from the genera *Bacillus* (*Bacillus agaradhaerens*, *Bacillus halodurans*, *Bacillus xiamenensis*, and *Bacillus cellulosilyticus*, respectively). The isolates BN01, BN03, and BN04 were from the genus *Nesterenkonia* (*Nesterenkonia alba*, *Nesterenkonia aethopica*, and *Nesterenkonia* sp. YIM, respectively). The last genus found was the *Alkalilimnicola* isolate BN05 (Table S7 [phylogenetic analysis and identification of the bacterial isolates isolated from soda lakes soil of the Kenyan Rift Valley]). Most of the isolates were associated with the *Bacillus* genera. *Bacillus* strain showed the highest nitrile hydratase activity, while *Alkalilimnicola* strain had the highest nitrilase activity from the Bertholet reaction.

## 4. Discussion

In this study, we successfully recovered BN- and IBN-degrading bacteria from soda lake samples. Morphologically, most of the isolates were white in color with yellow pigments. During characterization, we noted that these yellow pigments increased after sometime, possibly due to stress, age, and desiccation. IHamadi and Kachiru [25] and Kiplimo et al. [9] reported this condition from bacterial isolates they obtained from hypersaline environment. The bacterial isolates showed tolerance to various salt concentrations. The favorable growth was recovered at pH 9.0, suggesting that isolates were alkaliphiles. This corresponds to a study by Banciu and Sorokin [26], who reported that *Bacillus alkalinitricus* sp. isolated from soda solonchak soil enriched with IBN could grow optimally at pH 10.0. There are reports of halophilic bacteria from South African Saltpan with the ability to grow at pH 8.8 [27]. Duckworth et al. [28] isolated 122 haloalkaliphilic bacteria from saline system of Southern Tunisia with favorable growth at pH 10.0. These isolates tolerated various salt concentrations indicating their versatility and tolerance to a wide range of salinity. Studies on bacterial isolates from Lake Magadi have revealed that these isolates can thrive at salt concentrations below 5% but can survive a wide range of salt concentrations up to 30% [9]. Nyakeri et al. [29] isolated and characterized haloalkaliphilic bacteria from Kenyan soda lake Magadi that could withstand up to 20% salt concentration, though the optimum salt concentration was found to be between 5% and 10% which is consistent with this study. The results of a separate study from Lake Lonar in India revealed that isolates were extremely halotolerant, growing at 25% and pH range of 7.0–11.0 [30]. The bacterial isolates grew at temperature range of 28°C–45°C with favorable growth recorded at 35°C–45°C. Several strains of haloalkaliphilic bacteria have the ability to tolerate temperature range of 25°C–45°C [31].

Enzyme screening using pH indicator has been used to screen for nitrilase and nitrile hydratase enzymes [32]. Nitrile hydratase was the highly produced enzyme by eight isolates; hence, more amide was produced during indicator screening color change from blue to green [17]. During screening, the color of the nitrile hydratase changes from blue to green, whereas the color of the nitrilase enzyme changes from blue to yellow [17]. During nitrile hydrolysis, hydrogen ions are released, which causes a change in color [18, 33]. Nitrile is hydrolyzed by nitrilase to produce carboxylic acid, which decreases the pH, resulting in a virtually observable color change. Nitrilase-producing bacteria release carboxylic acid and ammonia. The ammonia released mixes with Nessler's reagent, causing a color change from colorless to orange. On the other hand, nitrile hydratases producing bacteria release an amide; no ammonia is released, and therefore, no color change occurs when mixed with Nessler's reagent [17]. Bertholet reaction was used to quantify the resultant enzymes by analyzing ammonia released, indicated by a blue color [34]. This color change is related to ammonia generated [20]. Our findings concurred with the findings by [20], who used 96 plates well based on the indicator. His reaction was also at pH 7.0 same as our pH reaction.

Nitrile wastes are mainly used by microorganism as a nutrient, thereby producing microbial metabolites [35]. Traditional nitrile degradation methods required harsh conditions and produced secondary pollutants. Microbial nitrile degradation offers an environmentally friendly and efficient alternative [36]. For instance, a study highlighted the use of recombinant microbes for the effective removal of waste from the environment, emphasizing the cost-effectiveness and safety of microbial metabolic processes over physicochemical approaches. Nitrile-degrading bacteria nitrilase and nitrile hydratase are used in detoxifying industrial nitrile wastewater. These compounds are used in the chemical, pharmaceutical, and agricultural industries and are toxic to both humans and the environment. Du et al., [37] studied *Rhodococcus erythropolis*, which is used for the biodegradation of nitrile pollutants in industrial effluents. Report by [38] on recombinant *Pseudomonas putida* strains enhances nitrilase activity by degrading industrial waste acrylonitrile to amide and acid. A study conducted by [27] reported on the efficacy of microbial enzymes to convert nitrile pollutants into less toxic amides and carboxylic acids by biocatalytic methods. Bacteria with nitrilase enzyme isolated from contaminated agricultural fields demonstrated the ability to break down pesticide residues [11]. Investigation of *Bacillus* sp. isolated from wastewater treatment plants [39] showed their ability to efficiently degrade cyanide from industrial wastes. Du et al., [37] reviewed genetically engineered nitrile hydratases that improve bioremediation in polluted environments.

An ecological perspective shows that nitrile-degrading bacteria contribute to nitrogen cycling and microbial diversity in natural and anthropogenic ecosystems. Lavrov et al. [40] studied *Arthrobacter* and *Rhodococcus* species impact on nitrile contamination on soil microbial communities in contaminated sites. Lin et al. [41] investigated nitrile-degrading bacteria in extreme environments, such as alkaline soda lakes, identifying new strains with potential industrial applications. Pei et al. [42] analyzed metagenomic data from nitrile-rich environments, showing how microbial consortia adapt to nitrile degradation and influence ecosystem functioning.

16S rRNA gene sequencing analysis and BLAST results clustered the bacteria isolates into *Bacillus*, *Nesterenkonia*, and *Alkalilimnicola* genera. Isolate BN02 had 98% similarity to *B. agaradhaerens*, Gram-positive rod-shaped bacterium with the ability to degrade nitrile through nitrilase enzyme. In recent literature, there is limited information on *B. agaradhaerens* strain MOLA1024. However, *Bacillus* species are recognized for their enzymatic activities in degrading industrial pollutants, including nitriles. Zhang et al. [43] reported on the isolation and characterization of a *B. agaradhaerens* MOLA1024 strain from industrial effluent. Although the study primarily focused on its overall biocatalytic potential, the authors observed significant nitrile hydratase activity, that is, strain's ability to convert aliphatic nitriles to amides under mild conditions. Optimal enzyme activity was observed at pH 8.0 and 35°C, suggesting its application in industrial waste streams management. The report emphasized the use of this strain in biocatalyst processes to detoxify nitrile-containing industrial wastes. Zhang et al. [43] highlighted that *B. agaradhaerens* enzymatic conversion could be a sustainable alternative to traditional chemical treatments. Shen et al. [44] reported that *B. agaradhaerens* had the ability to produce bioflocculants, which are used in microalgae harvest, heavy metal removal, and treatment of wastewater.

Isolates BN06, BN08 BN09, BN10, IBN12, and IBN13 had the closest relative to *B. halodurans* with BLAST similarity of 98%–99.98%, a rod shaped, Gram positive, facultative haloalkaliphilic, motile and spore-forming bacterium. This bacterium is known for the production of extracellular hydrolase [45]. The bacterium could degrade nitrile through an enzymatic reaction to produce an acid and ammonia. Therefore, it can potentially be used in waste treatment [45]. Bouacem et al. [46] characterized a halo-alkaline protease from *B. halodurans* strain US193, showing its stability across extreme conditions. The resilience in detergents and organic solvent degradation suggested its applications in industrial waste management processes. Investigation on *B. xiamenensis* role in bioremediation of heavy metal lead (Pb) demonstrated the bacterium's resistance to heavy metal contaminants, thus environmental biotechnology applications potential [47]. The research on *B. cellulosilyticus* DSM 2522, focusing on cellulolytic capabilities, emphasizes the role of lignocellulose degradation. The study provides insights into the bacterium's metabolic pathways, contributing to our understanding of microbial ecology in cellulose-rich environments [48].

Isolates BN01, BN03, and BN04 were closely related to *Nesterenkonia species* (*N. alba*, *Nesterenkonia aethiopica*, and *Nesterenkonia YIM90721*, respectively) from the BLAST results. *Nesterenkonia species* are Gram positive, rod-shaped, nonmotile, noncapsules forming and nonendospore-forming bacteria. There are reports on its ability to degrade specific nitrile [49]. *N. alba* is an alkaliphilic actinobacterium that grows in extreme environments. While there are limited specific studies on strains R-E 16S and RMR30, the species has been isolated from black liquor treatment systems in cotton pulp mills, showing its application in industrial waste management. Its ability to thrive in alkaline conditions suggests a role in degrading complex organic compounds, although direct evidence of nitrile degradation by these strains is not well-documented [49]. Specific information on *Nesterenkonia* sp. YIM 90721 is rare in recent publications. However, the genus has species with halophilic and alkaliphilic properties, thus applications in bioremediation of saline and alkaline industrial wastes. Further studies are necessary to determine the nitrile-degrading capabilities of the strain [49]. Isolates BN05 and BN07 from BLAST results had similarity to *Alkalilimnocola* genus phylum Proteobacteria. Alkalilimnicola means alkaline lake dwellers, supporting the finding that this isolate is a halophile [50] and has been studied for nitrogen and sulfur cycle roles. While direct evidence of strain AK92's involvement in nitrile degradation is not available, the genus's metabolic versatility indicates its potential application in environmental biotechnology in the bioremediation of alkaline environments.

Isolates IBN11 and IBN14 from BLAST results had similarity to genus *Bacillus* (*B. xiamenensis* and *B. cellulosityticus*), respectively. A patent describes *B. xiamenensis* strain capable of degrading bensulfuron-methyl, indicating pesticide degradation potential. However, specific information on nitrile degradation by strain APBSMLB19 is not detailed in recent literature. Yan et al. [39] investigated the *B. xiamenensis* isolated from soils near industrial areas with a wide range of pollutants to show their nitrile-degrading capacity. The strain demonstrated nitrile hydratase effectively catalyzing aromatic nitriles into less toxic acids. The enzyme operated efficiently at neutral pH and moderate temperatures based on kinetic analysis, which is advantageous for in situ bioremediation applications. Yan et al. proposed integration of these strains in bioaugmentation strategies to accelerate nitrile-contaminated site cleanup.

*B. cellulosilyticus* DSM 2522 has been investigated for its cellulolytic capabilities in alkaline conditions. Its role in lignocellulose degradation is known; however, there is no direct evidence of its involvement in nitrile degradation. Sun et al. [48] focused on the functional potential of *B. cellulosilyticus* DSM 252 to demonstrate its enzymatic activity beyond cellulolytic activity but also nitrile degradation. The strain was shown to degrade selected nitrile compounds with a focus on the role of nitrile hydratase and amidase enzymes to convert nitriles into corresponding acids. Bhatia et al. [38] experimented with extreme conditions and found that the strain maintained its activity, supporting its suitability in bioremediation integration. Kumar reported that deploying *B. cellulosilyticus* in waste management systems, especially in nitrile-containing wastes, enhances overall process efficiency and potential applications in industrial processes that require alkaline stability. These findings, therefore, are evidence that haloalkaliphilic group from the soda lake environment could be a source of novel nitrilases and hydratases enzymes. This may offer a more cost-effective solution when compared to traditional methods of degrading nitrile.

The enzymatic activities demonstrated in this study have notable industrial potential. Nitrilase and nitrile hydratase enzymes are already applied in multiple sectors, and the haloalkaliphilic strains identified here expand these opportunities. In the pharmaceutical industry, such enzymes can be used for the synthesis of optically pure carboxylic acids and amides, which are essential intermediates in drug production [44, 51, 52]. In the agrochemical sector, they offer a biocatalytic route for detoxifying nitrile-containing herbicides and pesticides, which is particularly relevant for regions surrounding soda lakes where agricultural runoff is common. In the food and fine chemical industries, nitrile-degrading enzymes can be used for biotransformation under alkaline conditions where conventional enzymes are unstable [53]. Moreover, their application in bioremediation is promising for the treatment of nitrile effluents from petrochemical, textile, and polymer industries [54]. From a scalability perspective, the ability of these enzymes to function under alkaline and saline conditions reduces the costs associated with high pH or salinity controls in industrial bioreactors. The robustness of haloalkaliphilic bacteria supports their integration into large-scale fermentation systems, and their potential immobilization on industrial carriers could further enhance stability and reusability. While commercial production would require optimization of fermentation processes and downstream purification, the feasibility of enzyme-based biocatalysis in continuous systems is well supported by their resilience to harsh environments. Thus, the isolates described here can form the basis for commercial enzyme production pipelines that align with green chemistry principles.

## 5. Conclusion

The study reported 14 isolates and 3 specific genus that can tolerate extreme conditions and present their potential nitrile industrial application. Strains that can degrade nitrile to acid and amide are not harmful to the environment but are useful to the green industry. Thus, the biodegradation of nitriles by bacteria from soda lakes can be considered as a promising direction in reducing the harm from the interaction of these toxic compounds with the environment. Enzymatic assays, colorimetric methods, Berthelot reaction, and colorimetric two-step nesslerization method are indispensable for isolation and further characterization of nitrile-degrading bacteria. However, several issues are still relevant in the study of nitrile biodegradation, even though there is a breakthrough. Such factors include the ability of the nitrile-degrading enzymes to function optimally under the industrial conditions, the possibilities of genetic engineering of the bacteria, and the possibility of incorporating the whole process of nitrile degradation into the existing systems of waste management. Future research should focus on the following: optimizing nitrile-degrading enzymes for specific applications and using techniques of genetic engineering along with synthetic biology and extending laboratory established findings to full scale processes more especially in processing industrial effluent and polluted areas. Looking at ways through which microbial consortia can enhance the degradation of nitriles, this can include better efficiency in conditions that could be suboptimal.

### 5.1. Limitation

Despite the promising findings, this study has limitations. The number of isolates characterized was relatively small, and thus, the results may not fully represent the breadth of nitrile-degrading microbial diversity present in Kenyan soda lakes. Enzyme assays were performed under controlled laboratory conditions, which do not always reflect the complexity of industrial environments. Additionally, proteomic analysis was not undertaken, limiting understanding of the genetic basis and regulation of nitrilase and nitrile hydratase production. Finally, pilot-scale validation of enzyme activity in real industrial effluents was beyond the scope of this work. Future studies should address these limitations by expanding the diversity of isolates tested, applying omics-based approaches, and conducting scale-up trials to assess true commercial feasibility.

## Data Availability

Data included in article/Supporting Information are readily available for sharing upon request.

## Supporting Information

Supporting Information Additional supporting information can be found online in the Supporting Information section. Table S1 Enrichment broth preparations. Table S2: Bacteriological agar preparation. Table S3: Morphological characterization. Table S4: Gram staining results. Table S5: The colorimetric test results. Table S6:The summary on the quantification and qualification by Nessler's reagent. Table S6A:The confirmatory test for nitrile enzymes. Table S7: Phylogenetic analysis. Figure S1: Quantitative analysis of nitrile hydratase producing bacteria. Figure S2: Quantitative analysis of nitrilase. Figure S3: The flowchart showing screening pipeline for confirmatory test with Nessler's reagent. Figure S3A: The confirmatory test for nitrile enzymes using Nessler's reagent. Figure S4: Bacterial growth at different pH values. Figure S5: Bacterial growth at different salt concentration. Figure S6: Bacterial growth at different temperatures.

## References

[1] Varrella S., Tangherlini M., Corinaldesi C. (2020). Deep Hypersaline Anoxic Basins as Untapped Reservoir of Polyextremophilic Prokaryotes of Biotechnological Interest. *Marine Drugs*.

[2] Sorokin D. Y., Berben T., Melton E. D., Overmars L., Vavourakis C. D., Muyzer G. (2014). Microbial Diversity and Biogeochemical Cycling in Soda Lakes. *Extremophiles*.

[3] Grant W. D., Jones B. E. (2016). Bacteria, Archaea and Viruses of Soda Lakes. *Soda Lakes of East Africa*.

[4] Espliego J. M. E., Saiz V. B., Torregrosa-Crespo J. (2018). Extremophile Enzymes and Biotechnology. *Extremophiles*.

[5] Schagerl M. (2016). Dipping into the soda lakes of East Africa. *Soda Lakes of East Africa*.

[6] Banciu H. L., Muntyan M. S. (2015). Adaptive Strategies in the Double-Extremophilic Prokaryotes Inhabiting Soda Lakes. *Current Opinion in Microbiology*.

[7] Kambura A. K., Mwirichia R. K., Kasili R. W., Karanja E. N., Makonde H. M., Boga H. I. (2016). Bacteria and Archaea Diversity Within the Hot Springs of Lake Magadi and Little Magadi in Kenya. *BMC Microbiology*.

[8] Kaplan O., Veselá A. B., Petříčková A. (2013). A Comparative Study of Nitrilases Identified by Genome Mining. *Molecular Biotechnology*.

[9] Kiplimo D., Mugweru J., Kituyi S., Kipnyargis A., Mwirichia R. (2019). Diversity of Esterase and Lipase Producing Haloalkaliphilic Bacteria From Lake Magadi in Kenya. *Journal of Basic Microbiology*.

[10] Orwa P., Mugambi G., Wekesa V., Mwirichia R. (2020). Isolation of Haloalkaliphilic Fungi From Lake Magadi in Kenya. *Heliyon*.

[11] Singh R. V., Sharma H., Koul A., Babu V. (2018). Exploring a Broad Spectrum Nitrilase From Moderately Halophilic Bacterium *Halomonas* sp. IIIMB2797 Isolated From Saline Lake. *Journal of Basic Microbiology*.

[12] Yang Y., Wei L., Cui L., Zhang M., Wang J. (2017). Profiles and Risk Assessment of Heavy Metals in Great Rift Lakes, Kenya. *Clean-Soil, Air, Water*.

[13] Egelkamp R., Zimmermann T., Schneider D., Hertel R., Daniel R. (2019). Impact of Nitriles on Bacterial Communities. *Frontiers in Environmental Science*.

[14] Mukram I., Nayak A. S., Kirankumar B., Monisha T. R., Reddy P. V., Karegoudar T. B. (2015). Isolation and Identification of a Nitrile Hydrolyzing Bacterium and Simultaneous Utilization of Aromatic and Aliphatic Nitriles. *International Biodeterioration and Biodegradation*.

[15] Ramteke P. W., Maurice N. G., Joseph B., Wadher B. J. (2013). Nitrile-Converting Enzymes: An Eco-Friendly Tool for Industrial Biocatalysis. *Biotechnology and Applied Biochemistry*.

[16] Thuku R. N., Brady D., Benedik M. J., Sewell B. T. (2009). Microbial Nitrilases: Versatile, Spiral Forming, Industrial Enzymes. *Journal of Applied Microbiology*.

[17] Sahu R., Meghavarnam A. K., Janakiraman S. (2019). A Simple, Efficient and Rapid Screening Technique for Differentiating Nitrile Hydratase and Nitrilase Producing Bacteria. *Biotechnology Reports*.

[18] Aziz M., TriWijayanta A., Nandiyanto A. B. D. (2020). Ammonia as Effective Hydrogen Storage: A Review on Production, Storage and Utilization. *Energies*.

[19] Stewart B. M. (1985). Effect of Temperature on the Formation of Indophenol Blue in a Spectrophotometric Method for the Determination of Ammonia. *Water Research*.

[20] Santoshkumar M., Nayak A. S., Anjaneya O., Karegoudar T. B. (2010). A Plate Method for Screening of Bacteria Capable of Degrading Aliphatic Nitriles. *Journal of Industrial Microbiology and Biotechnology*.

[21] Cappuccino J. G., Sherman N. (1996). *Instructor’s Guide for Microbiology: A Laboratory Manual*.

[22] Sambrook J., Fritsch E. F., Maniatis T. (1989). *Molecular Cloning: A Laboratory Manual*.

[23] Filipski A., Tamura K., Billing-Ross P., Murillo O., Kumar S. (2015). Phylogenetic Placement of Metagenomic Reads Using the Minimum Evolution Principle. *BMC Genomics*.

[24] Kumar S., Stecher G., Tamura K. (2016). MEGA7: Molecular Evolutionary Genetics Analysis Version 7.0 for Bigger Datasets. *Molecular Biology and Evolution*.

[25] IHamadi B., Kachiru R. (2015). Characterization, Enzymatic Activity, and Secondary Metabolites of Fungal Isolates From Lake Sonachi in Kenya. *IOSR Journal of Pharmacy and Biological Sciences*.

[26] Banciu H. L., Sorokin D. Y. (2013). Adaptation in Haloalkaliphiles and Natronophilic Bacteria. *Polyextremophiles: Life Under Multiple Forms of Stress*.

[27] Alluri N. R., Chandrasekhar A., Vivekananthan V. (2017). Scavenging Biomechanical Energy Using High-Performance, Flexible BaTiO_3_ Nanocube/PDMS Composite Films. *ACS Sustainable Chemistry and Engineering*.

[28] Duckworth A. W., Grant W. D., Jones B. E., Steenbergen R. (2006). Phylogenetic Diversity of Soda Lake Alkaliphiles. *FEMS Microbiology Ecology*.

[29] Nyakeri E. M., Mwirichia R., Boga H. (2018). Isolation and Characterization of Enzyme Producing Bacteria From Lake Magadi, an Extreme Soda Lake in Kenya. *Journal of Microbiology & Experimentation*.

[30] Joshi A., Thite S., Karodi P., Joseph N., Lodha T. (2021). *Alkalihalobacterium elongatum* gen. nov. sp. nov.: An Antibiotic-Producing Bacterium Isolated From Lonar Lake and Reclassification of the Genus *Alkalihalobacillus* Into Seven Novel Genera. *Frontiers in Microbiology*.

[31] Kambura A. K., Mwirichia R. K., Kasili R. W., Karanja E. N., Makonde H. M., Boga H. I. (2016). Diversity of Fungi in Sediments and Water Sampled From the Hot Springs of Lake Magadi and Little Magadi in Kenya. *African Journal of Microbiology Research*.

[32] Egelkamp R., Schneider D., Hertel R., Daniel R. (2017). Nitrile-Degrading Bacteria Isolated From Compost. *Frontiers in Environmental Science*.

[33] Banerjee A., Kaul P., Sharma R., Banerjee U. C. (2003). A High-Throughput Amenable Colorimetric Assay for Enantioselective Screening of Nitrilase-Producing Microorganisms Using pH Sensitive Indicators. *Journal of Biomolecular Screening*.

[34] Weatherburn M. W. (1967). Phenol-Hypochlorite Reaction for Determination of Ammonia. *Analytical Chemistry*.

[35] Martins R. F., Delgado O., Hatti-Kaul R. (2003). Sequence Analysis of Cyclodextrin Glycosyltransferase From the *Alkaliphilic Bacillus* agaradhaerens. *Biotechnology Letters*.

[36] Levy-Booth D. J., Prescott C. E., Grayston S. J. (2014). Microbial Functional Genes Involved in Nitrogen Fixation, Nitrification and Denitrification in Forest Ecosystems. *Soil Biology and Biochemistry*.

[37] Du W., Huang J., Cui B., Guo Y., Wang L., Liang C. (2021). Efficient Biodegradation of Nitriles by a Novel Nitrile Hydratase Derived from *Rhodococcus erythropolis* CCM2595. *Biotechnology and Biotechnological Equipment*.

[38] Bhatia R. K., Bhatia S. K., Mehta P. K., Bhalla T. C. (2014). Biotransformation of Nicotinamide to Nicotinyl Hydroxamic Acid at Bench Scale by Amidase Acyl Transfer Activity of *Pseudomonas putida* BR-1. *Journal of Molecular Catalysis B: Enzymatic*.

[39] Yan G., Zhang Y., Wang J. (2017). Recent Advances in the Synthesis of Aryl Nitrile Compounds. *Advanced Synthesis and Catalysis*.

[40] Lavrov K. V., Shemyakina A. O., Grechishnikova E. G., Novikov A. D., Kalinina T. I., Yanenko A. S. (2019). *In Vivo* Metal Selectivity of Metal-Dependent Biosynthesis of Cobalt-Type Nitrile Hydratase in *Rhodococcus* Bacteria: A New Look at the Nitrile Hydratase Maturation Mechanism?. *Metallomics*.

[41] Lin C., Shen Y., Huang B., Liu Y., Cui S. (2017). Synthesis of Amides and Nitriles From Vinyl Azides and *p*-Quinone Methides. *Journal of Organic Chemistry*.

[42] Pei X., Wu Y., Wang J. (2020). Biomimetic Mineralization of Nitrile Hydratase Into a Mesoporous Cobalt-Based Metal-Organic Framework for Efficient Biocatalysis. *Nanoscale*.

[43] Zhang Q. L., Liu Y., Ai G. M., Miao L. L., Zheng H. Y., Liu Z. P. (2012). The Characteristics of a Novel Heterotrophic Nitrification-Aerobic Denitrification Bacterium, *Bacillus methylotrophicus* Strain L7. *Bioresource Technology*.

[44] Shen J., Cai X., Liu Z., Zheng Y. (2021). Nitrilase: A Promising Biocatalyst in Industrial Applications for Green Chemistry. *Critical Reviews in Biotechnology*.

[45] Takami H., Nakasone K., Takaki Y. (2000). Complete Genome Sequence of the Alkaliphilic Bacterium *Bacillus halodurans* and Genomic Sequence Comparison With *Bacillus subtilis*. *Nucleic Acids Research*.

[46] Bouacem K., Amziane-Touazi M., Ben Hania W. (2022). Isolation and Characterization of Moderately Thermophilic Aerobic Cultivable Bacteria From Hammam Righa Hot Spring (Algeria): Description of Their Hydrolytic Capacities ARTICLE INFO ABSTRACT/RESUME. *Algerian Journal of Environmental Science*.

[47] Phengnoi P., Thakham N., Rachphirom T., Teerakulkittipong N., Lirio G. A., Jangiam W. (2022). Characterization of Levansucrase Produced by Novel *Bacillus siamensis* and Optimization of Culture Condition for Levan Biosynthesis. *Heliyon*.

[48] Sun J., Shi S., Wu J., Xie R., Geng A., Zhu D. C. (2016). Characterization of a Salt-Tolerant and Cold-Adapted Xylanase From *Bacillus cellulosilyticus*. *BioResources*.

[49] Luo H. Y., Wang Y. R., Miao L. H. (2009). *Nesterenkonia alba* sp. nov., an Alkaliphilic Actinobacterium Isolated From the Black Liquor Treatment System of a Cotton Pulp Mill. *International Journal of Systematic and Evolutionary Microbiology*.

[50] Richey C. (2008). *Probing the Novelties of Alkalilimnicola ehrlichii Strain MLHE-1 With Genomic and Proteomic Approaches*.

[51] Martínková L. (2019). Nitrile Metabolism in Fungi: A Review of Its Key Enzymes Nitrilases With Focus on Their Biotechnological Impact. *Fungal Biology Reviews*.

[52] Xue Y. P., Yang Y. K., Lv S. Z., Liu Z. Q., Zheng Y. G. (2016). High-Throughput Screening Methods for Nitrilases. *Applied Microbiology and Biotechnology*.

[53] Park J. M., Trevor Sewell B., Benedik M. J. (2017). Cyanide Bioremediation: The Potential of Engineered Nitrilases. *Applied Microbiology and Biotechnology*.

[54] Martínková L., Rucká L., Nešvera J., Pátek M. (2017). Recent Advances and Challenges in the Heterologous Production of Microbial Nitrilases for Biocatalytic Applications. *World Journal of Microbiology and Biotechnology*.

