# Supplementary material for: Novel Haloalkaliphilic Nitrile-Degrading Bacteria From Soda Lake Soil of Rift Valley Kenya
Source: ScientificWorldJournal. 2025 Nov 19;2025:3126129. doi: 10.1155/tswj/3126129 (PMC12657091; doi:10.1155/tswj/3126129)
Supplement: Supporting Information — Additional supporting information can be found online in the Supporting Information section. Table S1 Enrichment broth preparations. Table S2: Bacteriological agar preparation. Table S3: Morphological characterization. Table S4: Gram staining results. Table S5: The colorimetric test results. Table S6:The summary on the quantification and qualification by Nessler's reagent. Table S6A:The confirmatory test for nitrile enzymes. Table S7: Phylogenetic analysis. Figure S1: Quantitative analysis of nitrile hydratase producing bacteria. Figure S2: Quantitative analysis of nitrilase. Figure S3: The flowchart showing screening pipeline for confirmatory test with Nessler's reagent. Figure S3A: The confirmatory test for nitrile enzymes using Nessler's reagent. Figure S4: Bacterial growth at different pH values. Figure S5: Bacterial growth at different salt concentration. Figure S6: Bacterial growth at different temperatures. [file 3126129.f1.docx]

**Appendix**

| **Components** | **Quantity** |  |
| --- | --- | --- |
| Lake water | 1liter | Autoclaved at 121°C for 15 minutes, cooled to 45 °C and add 2µl of vitamin B_12_ |
| Isobutyronitrile | 2.5ml |  |
| Butyronitrile | 2.5ml |  |

Table A1: Enrichment broth preparation per liter of sterile lake water

Bacteriological agar plates were prepared as described in Table 2

| **Components** | **Quantity** |  |
| --- | --- | --- |
| Agar | 7.5 grams | Autoclaved at 121 °C for 15 minutes, cooled to 45 °C and add 2µl of vitamin B_12_ |
| Isobutyronitrile | 2.5ml |  |
| Butyronitrile | 2.5ml |  |
| Lake water | 250ml |  |

TableA2: Bacteriological agar plates preparation

This procedure was repeated for the preparation of butyronitrile plates.

| **Morphological characterization of the bacterial isolates** | | | | | | | | |
| --- | --- | --- | --- | --- | --- | --- | --- | --- |
| **Substrates/code** | **Sources** | **Color** | **Consistency** | **Texture** | **Margin** | **Size** | **Shape** | **Elevation** |
| BN01 | L.Magadi grassland soil | white cream | Mucoid | Smooth | Entire | Small | Irregular | Flat |
| BN02 | L.Magadi grassland soil | White | Mucoid | Rough | Serrated | Medium | Irregular | Growing into the medium |
| BN03 | L.Magadi grassland soil | white cream | Mucoid | Rough | Serrated | Small | Irregular | Growing off line of streaking |
| BN04 | L.Magadi grassland soil | White | Mucoid | Smooth | Entire | Small | Irregular | Growing into the medium |
| BN05 | L.Magadi grassland soil | white cream | Mucoid | Filamentous | Serrated | Medium | Irregular | Growing off line of streaking |
| BN06 | L.Magadi grassland soil | Cream | Mucoid | Rough | Serrated | Medium | Irregular | Flat |
| BN07 | L.Magadi grassland soil | cream | Mucoid | Smooth | Entire | Small | Irregular | Flat |
| BN08 | L.Magadi grassland soil | Cream/white | Mucoid | Rough | Serrated | Small | Irregular | Raised |
| BN09 | L.Magadi grassland soil | Cream/white | Mucoid | Smooth | Entire | Medium | Irregular | Flat |
| BN10 | L.Bogoria dry soil | yellow cream | Mucoid | Smooth | Entire | Very small | Rough | Concave |
| BN11 | L.Bogoria dry soil | Cream | Mucoid | Smooth | Serrated | Small | Irregular | Growing into the medium |
| BN12 | L.Bogoria dry soil | Yellow | Mucoid | Smooth | Entire | Small | Rough | Raised |
| BN13 | L.Bogoria dry soil | Yellow | Mucoid | Smooth | Entire | Very small | Rough | Raised |
| BN14 | L.Bogoria dry soil | Yellow | Mucoid | Smooth | Entire | Small | Rough | Raised |
| BN15 | L.Bogoria dry soil | White | Mucoid | Smooth | Entire | Very small | Rough | Raised |
| BN16 | L.Bogoria dry soil | Yellow | Mucoid | Smooth | Entire | Very small | Rough | Raised |
| BN17 | L.Bogoria dry soil | White | Mucoid | Smooth | Entire | Small | Rough | Raised |
| BN18 | L.Elementaita grassland soil | yellow cream | Mucoid | Smooth | Entire | Small | Rough | Concave |
| BN19 | L.Elementaita grassland soil | White | Mucoid | Smooth | Entire | Small | Rough | Flat |
| BN20 | L.Elementaita grassland soil | white cream | Mucoid | Smooth | Entire | Small | Rough | Concave |
| BN21 | L.Elementaita grassland soil | White | Mucoid | Smooth | Entire | Medium | Rough | Raised |
| BN22 | L. Sonachi dry soil | White | Mucoid | Smooth | Entire | Small | Irregular | Raised |
| BN23 | L. Elementaita dry soil | Cream | Mucoid | Smooth | Entire | Small | Irregular | Raised |
| BN24 | L. Elementaita dry soil | Orange | Mucoid | Smooth | Entire | Small | Rough | Raised |
| BN25 | L. Sonachi dry soil | White | Mucoid | Smooth | Entire | Small | Irregular | Raised |
| BN26 | L. Sonachi dry soil | Cream | Mucoid | Smooth | Entire | Small | Irregular | Raised |
| BN27 | L. Sonachi dry soil | White | Mucoid | Smooth | Entire | Small | Rough | Flat |
| BN28 | L. Sonachi grassland soil | White | Mucoid | Smooth | Entire | Small | Rough | Raised |
| BN29 | L. Sonachi grassland soil | yellow cream | Mucoid | Smooth | Entire | Small | Rough | Flat |
| BN30 | L. Sonachi grassland soil | Cream | Mucoid | Smooth | Entire | Small | Irregular | Flat |
| IBN31 | L. Magadi grassland soil | Cream/white | Dry/mucoid | Smooth | Serrated | Medium | Irregular | Flat |
| IBN32 | L. Magadi grassland soil | White/cream | Mucoid | Smooth | Entire | Medium | Irregular | Growing off line of streaking |
| IBN33 | L. Magadi grassland soil | Cream | Mucoid | Smooth | Entire | Medium | Irregular | Flat |
| IBN34 | L. Magadi grassland soil | Cream | Mucoid | Smooth | Entire | Small | Irregular | Flat |
| IBN35 | L. Magadi grassland soil | White | Mucoid | Smooth | Entire | Large | Irregular | Flat |
| IBN36 | L. Magadi grassland soil | Cream | Mucoid | Smooth | Entire | Small | Irregular | Flat |
| IBN37 | L. Magadi grassland soil | Cream | Mucoid | Smooth | Entire | Medium | Irregular | Flat |
| IBN38 | L. Magadi grassland soil | Cream | Mucoid | Smooth | Entire | Medium | Irregular | Flat |
| IBN39 | L. Bogoria Dry soil | White | Mucoid | Smooth | Entire | Very small | Irregular | Flat |
| IBN40 | L. Bogoria Dry soil | Yellow | Mucoid | Smooth | Entire | Small | Rough | Raised |
| IBN41 | L. Bogoria Dry soil | Cream | Mucoid | Smooth | Entire | Medium | Rough | Growing off line of streaking |
| IBN42 | L. Bogoria Dry soil | Yellow/cream | Mucoid | Smooth | Entire | Medium | Rough | Concave |
| IBN43 | L. Bogoria Dry soil | Yellow/cream | Mucoid | Smooth | Entire | Small | Rough | Growing off line of streaking |
| IBN44 | L. Elementaita grassland soil | Cream/white | Mucoid | Filamentous | Serrated | Medium | Irregular | Flat |
| IBN45 | L. Elementaita grassland soil | Cream/white | Mucoid | Filamentous | Serrated | Medium | Irregular | Flat |
| IBN46 | L. Elementaita grassland soil | Cream | Mucoid | Smooth | Entire | Very small | Rough | Raised |
| IBN47 | L. Elementaita grassland soil | Cream/white | Mucoid | Smooth | Entire | Medium | Irregular | Raised |
| IBN48 | L. Elementaita grassland soil | Cream/white | Mucoid | Smooth | Entire | Small | Rough | Raised |
| IBN49 | L. Elementaita dry soil | Cream/white | Mucoid | Smooth | Entire | Very small | Rough | Raised |
| IBN50 | L. Elementaita dry soil | White | Mucoid | Smooth | Entire | Small | Rough | Raised |
| IBN51 | L. Elementaita dry soil | White | Mucoid | Smooth | Entire | Small | Rough | Raised |
| IBN52 | L. Sonachi dry soil | White | Mucoid | Smooth | Entire | Small | Rough | Raised |
| IBN53 | L. Sonachi dry soil | White | Mucoid | Smooth | Entire | Small | Rough | Raised |
| IBN54 | L. Sonachi grassland soil | White | Mucoid | Smooth | Entire | Very small | Rough | Raised |
| IBN55 | L. Sonachi grassland soil | White | Mucoid | Smooth | Entire | Small | Rough | Raised |
| IBN56 | L. Sonachi grassland soil | White | Mucoid | Smooth | Entire | Small | Rough | Raised |
| IBN57 | L. Sonachi grassland soil | White | Mucoid | Smooth | Entire | Small | Rough | Raised |
| IBN58 | L. Sonachi grassland soil | Orange | Mucoid | Smooth | Entire | Small | Irregular | Raised |
| IBN59 | L. Bogoria Dry soil | Yellow | Mucoid | Smooth | Entire | Small | Rough | Flat |
| IBN60 | L. Bogoria Dry soil | Cream | Mucoid | Filamentous | Serrated | Medium | Irregular | Flat |

Table A3: Morphological characterization of the bacterial isolates based on butyronitrile and isobutyronitrile substrates

|  |  | |  | **Colony morphology** | | **Cell morphology** | | |
| --- | --- | --- | --- | --- | --- | --- | --- | --- |
| **S/NO** | **Medium** | | **Source** | **Color** | **Elevation** | **Cell arrangement** | **Gram stain** | **Shape** |
| 1 | BN | Lake Bogoria dry soil | | yellow cream | Convex | singled paired chain | Positive | Rod |
| 2 | BN | | Lake Bogoria dry soil | cream | growing into the medium | singled paired clustered | Positive | Rod |
| 3 | BN | | Lake Elementaita grassland soil | yellow cream | Convex | singled paired chain | Positive | Rod |
| 4 | BN | | Lake Elementaita grassland soil | white cream | Convex | singled paired chain | Positive | Rod |
| 5 | BN | | Lake Magadi grassland soil | white cream | Flat | singled paired clustered | negative | Rod |
| 6 | BN | | Lake Magadi grassland soil | White | growing into the medium | singled paired clustered | Positive | Rod |
| 7 | BN | | Lake Magadi grassland soil | white cream | growing off streaking line | singled paired clustered | negative | Rod |
| 8 | BN | | Lake Magadi grassland soil | White | growing into the medium | single paired clustered | Positive | Rod |
| 9 | BN | | Lake Magadi grassland soil | white cream | growing off streaking line | singled paired chain | Positive | Rod |
| 10 | BN | | Lake Sonachi grassland soil | yellow cream | flat | singled paired chain | Positive | Rod |
| 11 | IBN | | Lake Bogoria dry soil | cream | growing off the streak line | singled paired chain | Positive | Rod |
| 12 | IBN | | Lake Bogoria dry soil | yellow cream | convex | singled paired chain | Positive | Rod |
| 13 | IBN | | Lake Bogoria dry soil | yellow cream | growing off streaking line | singled paired chain | Positive | Rod |
| 14 | IBN | | Lake Magadi grassland soil | cream | growing off streaking line | singled paired chain | negative | Rod |

Table A4: The Gram staining results of the isolates that showed nitrilase and nitrile hydratase activity on indicator screening. All isolates BN01-IBN14 are rod shaped with IBN14, BN07, BN05 are Gram negative while BN01, BN02, BN03, BN04, BN06, BN08, BN09, BN10, IBN11, IBN12, IBN13 are Gram positive.

| **Indicator determination of enzyme activities** | | |
| --- | --- | --- |
| **Isolate code** | **Colour change** | **Enzymes** |
| BN01 | Yellow | Nitrilase |
| BN02 | Yellow | Nitrilase |
| BN03 | Green | nitrile hydratase |
| BN04 | Green | nitrile hydratase |
| BN05 | Yellow | Nitrilase |
| BN06 | Green | nitrile hydratase |
| BN07 | Green | nitrile hydratase |
| BN08 | Green | nitrile hydratase |
| BN09 | Yellow | Nitrilase |
| BN10 | Green | nitrile hydratase |
| BN11 | Yellow | Nitrilase |
| IBN12 | Yellow | Nitrilase |
| IBN13 | Green | nitrile hydratase |
| IBN14 | Green | nitrile hydratase |
| **Control** *E. coli* | no color change |  |
| Control BN | Yellow |  |
| Control IBN | Yellow |  |

Table A5: The colorimetric results of isolate BN01-IBN14. Nitrilase positive BN01, BN02, BN05, BN09, IBN11, IBN12, Nitrile hydratase positive BN03, BN04, BN06, BN07, BN08, BN10, IBN13 and IBN14, controls; E. coli negative control, IBN and BN positive control

| **Indicator determination of enzyme activities** | | | | | | |
| --- | --- | --- | --- | --- | --- | --- |
| **Isolate code** | **Colour change** | **Enzymes** | **Ammonia color** | **pH mean** | **Mean ammonia** | **Mean Berthot reaction** |
| BN01 | yellow | nitrilase | orange | 9.5 | 0.2075 | 0.173 |
| BN02 | yellow | Nitrilase | orange | 8.65 | 0.1685 | 0.108 |
| BN03 | green | nitrile hydratase | colourless | 9.35 | 0.1575 | 0.091 |
| BN04 | green | nitrile hydratase | colourless | 9.1 | 0.038 | 0.117 |
| BN05 | yellow | nitrilase | orange | 8.65 | 0.0715 | 0.094 |
| BN06 | green | nitrile hydratase | colourless | 9.7 | 0.0515 | 0.112 |
| BN07 | green | nitrile hydratase | colourless | 9.3 | 0.091 | 0.175 |
| BN08 | green | nitrile hydratase | colourless | 9.3 | 0.034 | 0.14 |
| BN09 | yellow | nitrilase | orange | 9.65 | 0.025 | 0.156 |
| BN10 | green | nitrile hydratase | colourless | 9.05 | 0.0475 | 0.18 |
| BN11 | yellow | nitrilase | orange | 9.45 | 0.1075 | 0.14 |
| IBN12 | yellow | nitrilase | orange | 8.95 | 0.1075 | 0.154 |
| IBN13 | green | nitrile hydratase | colourless | 8.7 | 0.151 | 0.496 |
| IBN14 | green | nitrile hydratase | colourless | 9.3 | 0.2905 | 0.714 |
| control *E. coli* | no color change | | no color change | 9.8 |  |  |
| control BN | yellow |  | yellow | 10 |  |  |
| control IBN | yellow |  | yellow | 10 |  |  |
| Ammonium chloride |  |  |  |  |  | 0.113 |

**Table A6:** The summary on the quantification and qualification of nitrilase and nitrile hydratase enzymes by use of Nessler’s reagent and nesslerization methods. Nitrilase positive BN01, BN02, BN05, BN09, IBN11, IBN12, Nitrile hydratase positive BN03, BN04, BN06, BN07, BN08, BN10, IBN13 and IBN14, controls; *E. coli* negative control, IBN and BN positive controls. Ammonium chloride for calibration.

| **Indicator and ammonia determination of nitrilase and nitrile hydratase activities**  **(Nesslerization method)** | | | | | |
| --- | --- | --- | --- | --- | --- |
| **Isolate code** | **pH mean** | **Control blank** | **Enzymes** | **Ammonia color** |  |
| BN01 | 9.5 | 0 | nitrilase | orange |  |
| BN02 | 8.65 | 0 | Nitrilase | orange |  |
| BN03 | 9.35 | 0 | nitrile hydratase | colourless |  |
| BN04 | 9.1 | 0 | nitrile hydratase | colourless |  |
| BN05 | 8.65 | 0 | nitrilase | orange |  |
| BN06 | 9.7 | 0 | nitrile hydratase | colourless |  |
| BN07 | 9.3 | 0 | nitrile hydratase | colourless |  |
| BN08 | 9.3 | 0 | nitrile hydratase | colourless |  |
| BN09 | 9.65 | 0 | nitrilase | orange |  |
| BN10 | 9.05 | 0 | nitrile hydratase | colourless |  |
| BN11 | 9.45 | 0 | nitrilase | orange |  |
| IBN12 | 8.95 | 0 | nitrilase | orange |  |
| IBN13 | 8.7 | 0 | nitrile hydratase | colourless |  |
| IBN14 | 9.3 | 0 | nitrile hydratase | colourless |  |
| control E. coli | 9.8 | 0 |  | no color change |  |
| control BN | 10 | 0 |  | orange |  |
| control IBN | 10 | 0 |  | orange |  |

**Table A6A**: The confirmatory test for nitrilase and nitrile hydratase enzyme using Nessler’s reagent. From BN1- IBN 14. Nitrilase positive BN01, BN02, BN05, BN09, IBN11, IBN12, Nitrile hydratase positive BN03, BN04, BN06, BN07, BN08, BN10, IBN13 and IBN14, controls; *E. coli* negative control, blanks of BN and IBN positive controls**.**

| **Molecular characterization** | | | | | |
| --- | --- | --- | --- | --- | --- |
| **Codes** | **Substrate** | **Soil source** | **Isolates** | **Closest strain** | **Identity %** |
| BN01 | Butyronitrile | Bogoria dry soil | Bacteria | *Nesterenkonia alba strain R-E 16S* | 99.51 |
| BN02 | Butyronitrile | Bogoria dry soil | Bacteria | *Bacillus agaradhaerens strain MOLA1024* | 99.88 |
| BN03 | Butyronitrile | Elementaita grassland soil | Bacteria | *Nesterenkonia alba strain RMR30* | 100.00 |
| BN04 | Butyronitrile | Elementaita grassland soil | Bacteria | *Nesterenkonia sp. YIM 90721* | 99.88 |
| BN05 | Butyronitrile | Magadi grassland soil | Bacteria | *Alkalilimnicola sp. AK92* | 92.87 |
| BN06 | Butyronitrile | Magadi grassland soil | Bacteria | *Bacillus halodurans strain BW411* | 99.39 |
| BN07 | Butyronitrile | Magadi grassland soil | Bacteria | *Alkalihalodubacillus halodurans* | 99.45 |
| BN08 | Butyronitrile | Magadi grassland soil | Bacteria | *Bacillus halodurans strain* | 99.77 |
| BN09 | Butyronitrile | Magadi grassland soil | Bacteria | *Bacillus halodurans strain* | 89.99 |
| BN10 | Butyronitrile | Sonachi grassland soil | Bacteria | *Bacillus halodurans strain US193* | 99.77 |
| IBN11 | Isobutyronitrile | Bogoria dry soil | Bacteria | *Bacillus xiamenensis APBSMLB19* | 100.00 |
| IBN12 | Isobutyronitrile | Bogoria dry soil | Bacteria | *Bacillus halodurans strain US193* | 99.97 |
| IBN13 | Isobutyronitrile | Bogoria dry soil | Bacteria | *Bacillus halodurans strain* | 99.97 |
| IBN14 | Isobutyronitrile | Magadi grassland soil | Bacteria | *Bacillus cellulosilyticus DSM 2522* | 98.37 |

**Table A7**: Phylogenetic analysis and identification of the bacterial isolates isolated from soda lakes soil of the Kenyan Rift Valley

**Figure A1:** Quantitative analysis of nitrile hydratase producing bacteria (BN3, BN4, BN6, BN7, BN8, BN10, IBN13 and IBN14) with turbid color due to production of amide.

**Figure A2;** Quantitative analysis of nitrilase. Nitrilase producing bacteria (BN1, BN2, BN5, BN9, IBN11 and IBN12) with blue color change due to production of carboxylic acid indicating presences of nitrilase enzyme.

Measure absorbance at 540nm (spectrophotometric quantification)

Calibration we used ammonium at 0.113nm, detection limit was based on incubation period.

Reaction with ammonia forms colourless – orange complex

Add Nessler’s reagent

Incubate with BN/IBN substrate minimal medium

Observe color change: Blue-yellow=nitrilase, Blue -green= nitrile hydratase

Isolate inoculation in TSB +BN/IBN substrate

Incubation at 30 ̊C, 18hrs, 115rpm

Add bromothymol blue (BTB) pH indicator (1%)

Figure A3: A flowchart showing screening pipeline for the confirmatory test by Nessler’s reagent. The pipeline was the same for other screening.

**Figure A3A**: The confirmatory test for nitrilase and nitrile hydratase enzyme using Nessler’s reagent. From BN1- IBN 14. Nitrilase positive BN01, BN02, BN05, BN09, IBN11, IBN12, Nitrile hydratase positive BN03, BN04, BN06, BN07, BN08, BN10, IBN13 and IBN14, controls; *E. coli* negative control, blanks of BN and IBN positive controls**.**

**Figure A4**: Bacterial growth at different pH values

**Figure A5**: Bacterial growth at different salinity levels

**Figure A6**: Bacterial growth at different temperatures
